# Comparison of Porosity and Thermal Conductivity of Concrete and Alkali-Activated Hybrid Binders in 3D-Printed Fiber-Reinforced Foamed Composites

**DOI:** 10.3390/ma18194498

**Published:** 2025-09-27

**Authors:** Magdalena Rudziewicz, Marcin Maroszek, Marek Hebda

**Affiliations:** Faculty of Materials Engineering and Physics, Cracow University of Technology, Warszawska 24, 31-155 Kraków, Poland; magdalena.rudziewicz@doktorant.pk.edu.pl (M.R.); marcin.maroszek@doktorant.pk.edu.pl (M.M.)

**Keywords:** foamed materials, 3D concrete printing, fiber reinforcement

## Abstract

Fiber-reinforced foamed composites have recently attracted growing interest due to their potential in sustainable construction and advanced additive manufacturing. However, their performance strongly depends on the type of matrix and fiber system used. The aim of this study was to perform a comparative analysis of matrix type and fiber composition on the porosity, thermal behavior, and mechanical performance of 3D-printed fiber-reinforced foamed composites. To this end, cementitious mixtures (M1–M3) were compared with alkali-activated hybrid binder systems (M4–M6). The results revealed marked differences in mechanical strength, dimensional stability, moisture transport, and interlayer cohesion. Alkali-activated specimens, particularly M5 and M6, exhibited superior compressive, flexural, and shear strength; reduced water penetration; and improved fiber–matrix bonding, associated with a denser and more homogeneous pore structure. In contrast, cementitious composites showed greater dimensional stability and easier process control, indicating practical advantages for large-scale on-site applications. The results highlight that while alkali activation and hybrid fiber reinforcement enhance structural performance, non-activated foamed concretes remain promising for applications prioritizing simplicity, reproducibility, and thermal insulation.

## 1. Introduction

The cement industry is undergoing a profound transformation by incorporating industrial and construction-derived waste materials, in response to both regulatory pressure and environmental concerns [1,2,3].

The substitution of virgin raw materials with by-products such as fly ash, ground granulated blast furnace slag (GGBFS), calcined clays, or crushed brick contributes to resource conservation and reductions in landfill burdens [4,5]. Supplementary cementitious materials (SCMs) not only contribute to the reduction in the carbon footprint associated with cement production but also enhance the physico-mechanical and functional performance of concrete. In particular, modifications in thermal conductivity imparted by SCM incorporation are of critical relevance for the development of energy-efficient building envelopes [6,7]. Binders containing recycled materials and alkali-activated systems synthesized from aluminosilicate-rich industrial by-products have been identified as promising low-emission alternatives to Portland cement, offering favorable mechanical and durability characteristics [8,9]. Industrial implementations further demonstrate the potential of recycling technologies, where construction and demolition waste is valorized into high-performance aggregates and binders [10,11].

Although industrial interest and extensive research efforts have increasingly focused on cementitious and geopolymer composites incorporating recycled constituents [12,13], there remains a lack of readily available material systems explicitly optimized for extrusion-based additive manufacturing. A discernible research gap persists with regard to 3D-printed foamed composites capable of simultaneously fulfilling all critical requirements, namely rheological performance and setting kinetics suitable for precision layer-by-layer deposition; sufficient interlayer cohesion; long-term durability under cyclic environmental exposure; and the effective incorporation of secondary raw materials such as fly ash, slag, or crushed brick waste. The present investigation seeks to address this multifaceted challenge by providing a direct comparative assessment of cementitious and alkali-activated foamed composites reinforced with a hybrid system of dispersed fibers.

In the face of accelerating urbanization and the globally increasing demand for cement, growing attention is being directed toward the substitution of conventional raw materials with recycled alternatives [14], including fly ash, blast furnace slag [15], and construction and demolition waste (CDW) [16,17]. Simultaneously, the rising stringency of energy efficiency standards for buildings [18] and the urgent need to reduce greenhouse gas emissions have heightened the importance of thermally insulating construction materials that align with the principles of the circular economy [19,20,21,22]. In this context, concrete incorporating recycled additives and alkali-activated-based or geopolymer-based composites demonstrate considerable potential as sustainable insulation materials. Their porous internal microstructure, inherently low thermal conductivity, and high fire resistance render them well-suited for use in energy-efficient and environmentally responsible construction applications [23,24,25]. The pursuit of sustainable and durable construction materials has led to diverse strategies, ranging from fiber reinforcement in foamed concretes to hybrid confinement systems for seawater sea-sand concrete. For example, Fu et al. [26] reported that GFRP–steel composite tube confinement can enhance the bearing capacity of concrete in offshore environments. While differing in scale and application, both approaches highlight the potential of hybrid binder–fiber systems to advance the mechanical reliability of next-generation composites. The use of such materials not only opens new avenues for sustainable building design but also contributes to the conservation of natural resources and a reduction in landfill waste generation.

Within the construction sector, alkali-activated or geopolymer foams are particularly promising as insulation materials due to their robust resistance to environmental factors, enabling their application across a wide range of climatic conditions [27,28]. A substantial portion of recent research has focused on the development and characterization of alkali-activated or geopolymer foams for thermal insulation purposes. Furthermore, ongoing technological advances are positioning alkali-activated or geopolymer materials as viable alternatives to conventional systems in a range of industrial applications. Among the most rapidly evolving innovations is the adoption of 3D printing with concrete, alkali-activated binders, geopolymers, and geopolymer composites, representing a transformative development in the traditionally conservative construction industry [29,30].

Additive manufacturing (AM) in construction not only enables highly customized architectural forms but also supports the principles of sustainable building by minimizing material waste, reducing formwork, and enhancing design efficiency. As such, 3D printing technologies align well with global policy frameworks prioritizing green, low-emission, and resource-efficient construction solutions. While the literature extensively covers the thermal performance criteria and fire resistance of conventional concrete systems, there remains a notable research gap concerning these parameters in 3D-printed structures [31,32]. This is particularly important since exterior walls account for about 25% of total heat loss. The development of sustainable insulation materials from renewable resources and industrial by-products, tailored for 3D printing, further increases their potential for future-oriented construction.

Porous geopolymer materials are characterized by exceptionally high porosity, often exceeding 70%, which contributes significantly to their low bulk density and advantageous thermal insulation properties [33]. These materials typically exhibit densities ranging from 0.30 to 0.80 g/cm^3^, rendering them substantially lighter than conventional Portland cement-based concretes [34]. Their thermal conductivity is correspondingly low, generally falling within the range of 0.05 to 0.48 W/(m·K), making them highly effective as thermal insulators [35,36,37]. The compressive strength of porous geopolymers typically varies between 0.2 and 8.13 MPa. While this is markedly lower than that of traditional structural concretes, it remains adequate for a wide spectrum of non-load-bearing and specialized applications [38,39,40,41]. These materials have demonstrated promising functionality in diverse sectors, notably in construction, where they serve as passive fire-protection elements due to their thermal stability and fire resistance.

In industrial contexts, porous geopolymers are also explored as catalytic supports, e.g., in biodiesel synthesis benefiting from their high surface area and thermal stability [42,43,44]. In the building and insulation sectors, porous alkali-activated binders or geopolymer composites can be utilized as multifunctional coatings that provide both thermal and acoustic insulation, thus improving energy efficiency and interior acoustic performance [43]. Among the critical factors influencing their thermal behavior is fiber reinforcement.

The incorporation of dispersed fibers has been shown to significantly reduce thermal conductivity, while also enhancing the ductility and reducing the brittleness of the composite matrix [45,46,47]. For instance, Walbrueck et al. observed that increasing fiber diameter from 125 to 250 µm resulted in a decrease in thermal conductivity from 0.0985 W/(m·K) to 0.0686 W/(m·K), which was attributed to more effective thermal barrier formation due to the spatial distribution of larger fibers within the geopolymer matrix [48]. Additional studies by the present authors demonstrated that the lowest thermal conductivity value, 0.056 W/(m·K), was achieved in a composite containing 45 wt% Miscanthus fibers (250 µm in diameter) and 0.35 wt% foaming agent. These fibers contribute to porosity via their internal cellular structure (parenchyma) and hydrophilic nature, which promotes pore formation at the fiber–matrix interface [49]. Agustini et al. reported that geopolymer foams reinforced with polypropylene (PP) fibers exhibited thermal conductivities as low as 0.2 W/(m·K) [50]. Similarly, composites reinforced with polyvinyl alcohol (PVA) and basalt fibers yielded thermal conductivities of 0.30 W/(m·K) and 0.38 W/(m·K), respectively [51]. Kozub et al. demonstrated that the addition of 1 wt% cotton fibers to 3D-printed fly ash-based geopolymer composites reduced thermal conductivity by approximately 12%, from 0.26 W/(m·K) to 0.23 W/(m·K), thereby improving the thermal insulation performance [52]. Research by Łaźniewska-Piekarczyk et al. evaluated the impact of recycled mineral wool (glass and rock wool) on the thermal conductivity of geopolymer matrices. The average thermal conductivity reached 1.053 W/(m·K) for glass wool and 0.953 W/(m·K) for rock wool [53]. In contrast, lightweight geopolymer foams incorporating 3 wt% and 5 wt% waste glass wool demonstrated significantly lower conductivities of 0.117 W/(m·K) and 0.113 W/(m·K), respectively [54].

Recent years have seen growing scientific interest in the utilization of hybrid fibers combinations of natural and synthetic fibers in alkali-activated binders or geopolymer composite systems. This approach is motivated by the potential of hybrid reinforcements to improve ductility, fracture resistance, and mechanical robustness. While previous studies have primarily focused on single-fiber systems, emerging research increasingly emphasizes the synergistic effects of hybrid fiber blends, which may provide superior performance compared to monofiber reinforcement [55]. As highlighted by Yang et al. [56] the rheological behavior of cement-based fiber-reinforced materials is a critical factor governing the entire cycle of three-dimensional concrete printing (3DCP), directly influencing both the printability and the structural quality of the fabricated elements. In the context of foamed alkali-activated binders or geopolymer composites, hybrid fiber reinforcement within the porous matrix appears to be a promising strategy for simultaneously enhancing mechanical and functional properties. For example, it has been demonstrated that geopolymer composites reinforced with a combination of steel and carbon fibers exhibited thermal conductivities in the range of 0.18–0.22 W/(m·K), while maintaining low density and adequate mechanical resistance. The fibers enhanced structural integrity without significantly compromising thermal insulation [57]. Particularly in additive manufacturing (AM) applications, hybrid fibers may facilitate pore stabilization, improve the matrix-fiber interface, and mitigate microcrack initiation and propagation. This makes them attractive candidates for the design of lightweight, durable, and thermally insulating foamed alkali-activated binders or geopolymer composites suitable for 3D printing.

Although substantial progress has been achieved in the development of recycled concrete and alkali-activated binders or geopolymer-based materials, the construction industry still lacks widely available, ready-to-use formulations tailored to the requirements of additive manufacturing. Increasing regulatory and environmental pressures, combined with rising demand for high-performance and energy-efficient housing, underscore the need for accessible, scalable, and sustainable material systems. Despite promising laboratory-scale results, foamed alkali-activated binders, geopolymer composites, and foamed concretes with recycled constituents require further process optimization to satisfy the technical specifications and operational constraints of large-scale 3D printing. The literature review identifies a research gap concerning the thermal conductivity of 3D-printed structural elements produced from alkali-activated binder composites, particularly those reinforced with the novel combination of glass and merino wool fibers, which remains largely unexplored. The present study addresses this knowledge gap by evaluating the thermal and mechanical performance of 3D-printed foamed concretes and alkali-activated binders, both incorporating recycled additives and reinforced with hybrid fiber systems.

## 2. Materials and Methods

### 2.1. Materials

**Figure 1:**
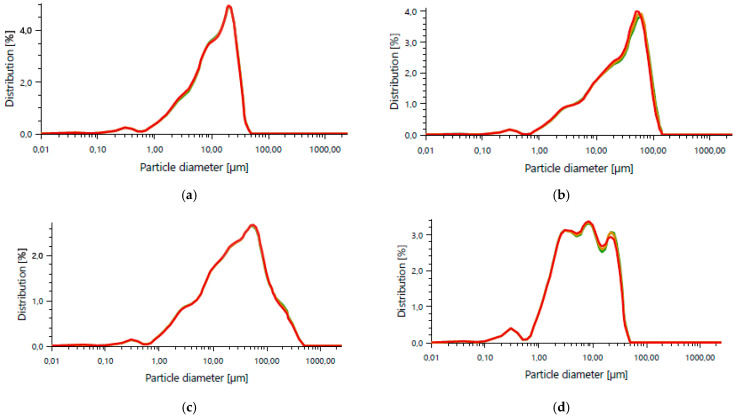
Particle size distribution: (**a**) CEM IV 42.5 cement, (**b**) fly ash, (**c**) coal slag, (**d**) ground demolition brick.

The primary base materials used in the experimental program were quartz sand and low-carbon cement CEM IV/B(V) 42.5N LH/NA, supplied by Holcim Group, Małogoszcz Cement Plant (Małogoszcz, Poland). Recycled components included ground demolition brick, fly ash sourced from PGE Energia Ciepła S.A. (Kraków, Poland), and coal slag obtained from Łęczyńska Energetyka Spółka z o.o. (Puchaczów, Poland). The particle size distribution determined using an Anton Paar PSA 1190 LD laser diffraction particle size analyzer (Graz, Austria) is presented in Figure 1.

Fly ash and coal slag exhibit a high proportion of alumina and silica, imparting pronounced pozzolanic activity, whereas sand constitutes an almost pure siliceous mineral filler. In contrast, CEM IV 42.5 cement is characterized by a substantial CaO content together with the presence of SO_3_, which are primarily responsible for its binding capacity and hydraulic performance. The chemical composition of the primary raw materials CEM IV 42.5 cement, fly ash and coal slag has been comprehensively characterized in the authors’ previous research [58]. In the present study, finely ground brick, sourced from construction and demolition waste, was additionally incorporated as a recycled component to enhance the sustainability profile of the composite formulations (Table 1).

**Figure 2:**
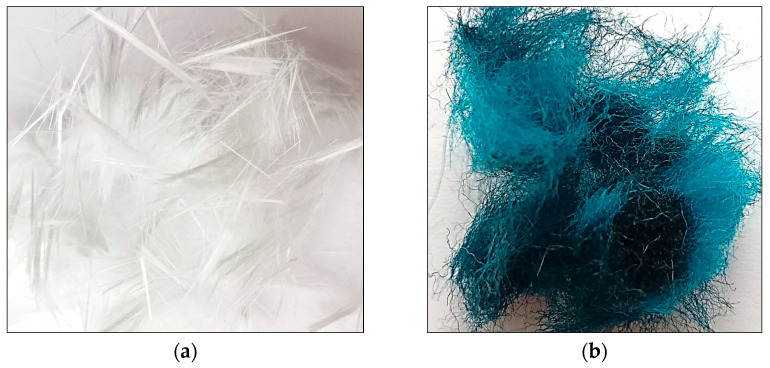
Representative view of (**a**) glass (G) and (**b**) merino wool (M) fibers.

To enhance the composite structure, a reinforcing blend of glass fibers and merino wool fibers was used, both derived from post-industrial waste. The characteristics of the fibers were presented in Figure 2 and Table 2.

As a foaming agent, a 5% synthetic surfactant (PIANOTWÓR, AS, MEEX AG, Chrzanów, Poland) was used, with the foam added to the mix at a 1:1 volumetric ratio. The alkaline activator solution used for the geopolymerization process consisted of 10 M sodium hydroxide and sodium silicate (water glass), combined at a molar ratio of 2.5 and a density of 1.45 g/cm^3^. A total of six fiber-reinforced foamed alkali-activated mixtures were prepared. The selection of fiber dosages and configurations was guided by findings from previous literature reviews and supported by the outcomes of the authors’ earlier experimental studies [58,59]. Their detailed compositions were presented in Table 3.

The preparation of the mixtures was conducted via two distinct approaches, due to the differing characteristics of the materials involved. For alkali-activated binder-based composites, the process commenced with the homogenization of fly ash and furnace slag, followed by the addition of an alkaline solution. The components were mixed for 15 min in a low-speed mixer (30 rpm) to ensure thorough blending. Once a homogeneous paste was obtained, a fiber-reinforced mortar was incorporated, followed by the gradual introduction of foam. The foam was added incrementally to avoid disrupting the integrity of the air bubble structure. The process concluded with controlled curing of the mixture in molds, without the application of mechanical compaction. In the case of concrete incorporating recycled additives, it was essential to first prepare a dry mixture containing the fiber reinforcement, and separately, a stable foam with strictly defined physical parameters. These two components were combined only after individual assessment of their physical properties, such as foam density and homogeneity. The integration of foam into the concrete matrix had to be carried out under conditions that ensured the overall stability of the mixture and prevented segregation. Following the combination of components, flowability tests and stability assessments were performed. If the mixture did not meet the required performance criteria, process parameters were adjusted accordingly. In both cases, the objective was to obtain a homogeneous, lightweight composite mixture with appropriate functional properties. Final specimens were evaluated for their applicability in 3D printing technology, particularly in terms of their ability to retain shape post-extrusion. Detailed procedures for mixture preparation have been described in the authors’ previous studies [58,59].

### 2.2. Methods

#### 2.2.1. Shrinkage Test

The shrinkage behavior of the 3D-printed concrete was assessed by measuring the linear dimensional changes in the printed specimens over time. The initial length of each specimen was recorded immediately after the printing process (time 0), serving as the reference measurement. Subsequent measurements were taken after 1 day, 14th day, 28th day to evaluate the time-dependent shrinkage. The specimens were cured under controlled laboratory conditions, at 22 ± 2 °C with a relative humidity of 55% ± 5%, to ensure consistent controlled environmental exposure and to facilitate reliable monitoring of shrinkage development over time. The methodology was intended primarily as a comparative assessment between the different material systems. At each testing interval, the alkali-activated mortars were weighed and their dimensions recorded. The principal objective of this test was to provide a preliminary indication of deformation tendencies, supporting the evaluation of material performance and informing subsequent investigations aimed at large-scale additive manufacturing applications. At present, there are no standardized procedures dedicated to shrinkage determination in 3D-printed cementitious composites. Linear shrinkage was calculated as the relative change in length with respect to the initial measurement, according to the following Formula (1):

(1) $$\varepsilon_{sh}=\frac{L_{0}-L_{t}}{L_{0}}\;\times 100\%$$

where

*ε_sh_*—linear shrinkage [%],
*L*_0_—initial length of the specimen (immediately after printing) [mm],
*L_t_*—length of the specimen after time t = 1, 14, 28 days) [mm].

#### 2.2.2. Flexural and Compressive Strength Testing Procedures

The evaluation of mechanical properties was performed using the MTS Criterion 43 universal testing system (MTS Systems Corporation, Eden Prairie, MN, USA), integrated with the MTS TestSuites 1.0 analytical software to ensure precise data acquisition and control. Flexural strength characterization was conducted on prismatic specimens with nominal dimensions of 40 × 40 × 160 mm, in strict accordance with the PN-EN ISO 178 standard. The three-point bending configuration employed a support span of 100 mm, with a constant crosshead displacement rate of 5 mm/min and three replicate measurements were performed for each specimen type to ensure statistical reliability.

Compressive strength testing followed the EN 12390-3 specification, utilizing cube specimens with edge lengths of 40 mm. The loading rate was set to a constant crosshead speed of 1 mm/min, until ultimate failure. Both tests were conducted on specimens after 14 and 28 days of curing with six measurements performed for each curing age to ensure statistical reliability.

#### 2.2.3. Thermal Conductivity Testing Procedures

The thermal insulation properties of the tested materials, specifically the thermal conductivity coefficient (λ) and thermal resistance (R), were determined using the HFM 446 Lambda heat flow meter, manufactured by Netzsch (Selb, Germany). Measurements were carried out based on the dual-plate method (hot and cold plate technique), in accordance with internationally recognized thermal metrology standards, including ASTM C1784, ASTM C518, ISO 8301, and EN 12664. Specimens were prepared as flat plates with dimensions of 200 × 200 × 50 mm.

#### 2.2.4. Thermographic Moisture Assessment of the Material

**Figure 3:**
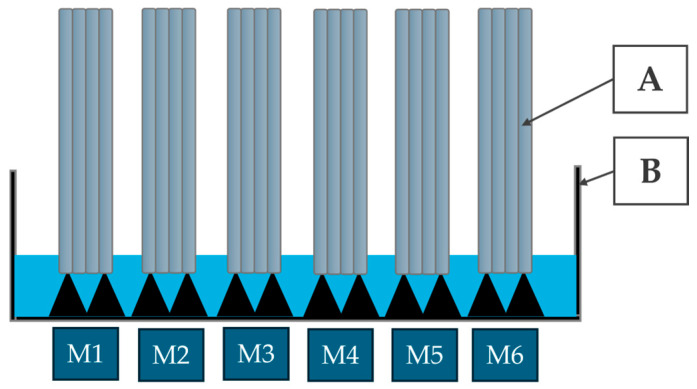
Schematic illustration of capillary water absorption test for thermographic moisture analysis: (**A**) foamed 3D-printed sample; (**B**) capillary tray.

Surface temperature measurements of the samples were conducted using a thermal imaging camera, FLIR E96 (Teledyne FLIR, Wilsonville, OR, USA), at predefined 5 min intervals over a 120 min observation period. This approach enabled monitoring of the dynamic changes associated with moisture migration through the 3DCP samples as a function of their composition. Cuboid specimens with standardized dimensions of 40 × 40 × 160 mm were evaluated using a partial immersion method. Each specimen was positioned vertically on a plastic mesh within shallow containers filled with water, ensuring consistent contact between the liquid and the designated suction surface (base area: 40 × 40 mm = 1600 mm^2^), while preventing full submersion [60,61]. The samples were placed in water with an immersion depth of 3mm. Water absorption progressed along the vertical axis of the specimen, in accordance with established capillary rise testing procedures (Figure 3). Water absorption due to capillary action was evaluated following the guidelines of EN 1015-18:2002 [62].

On the thermograms, regions with elevated moisture content appeared as cooler zones (e.g., blue), in contrast to drier areas, which exhibited higher surface temperatures (e.g., red or yellow).

#### 2.2.5. Layer Adhesion Tests

**Figure 4:**
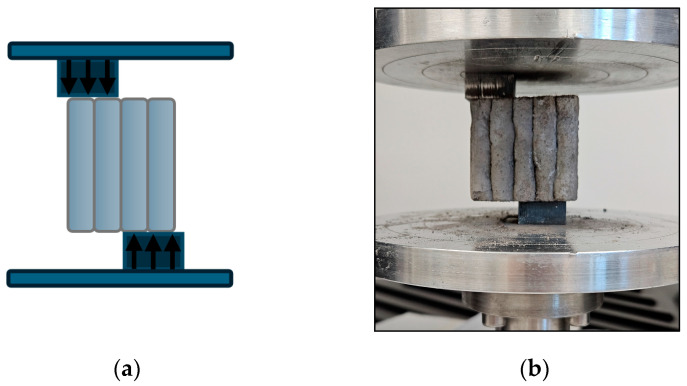
Schematic representation of the interlayer bond shear test (**a**) and specimen under testing (**b**).

To determine the interlayer shear strength, specimens produced from 3D-printed material were mounted in a universal testing machine according to the diagram shown in Figure 4. This arrangement generates a pure shear stress state along the interface between adjacent layers. The load was progressively increased, with a constant crosshead displacement rate of 1 mm/min, until bond failure occurred, indicated by relative layer displacement or complete separation of the interface. The maximum load at failure was recorded, and the shear strength is calculated as the ratio of this load to the bonded interface area, in accordance with previously described procedures [63]. Six independent measurements were carried out to ensure the reproducibility of the results, employing an MTS Criterion 43 universal testing machine (MTS Systems Corporation, Minnesota, USA).

#### 2.2.6. Microscopy Analysis

The surface morphology of the samples was investigated with a digital optical microscope: Keyence VHX-7000, KEYENCE International, Mechelen, Belgium. The use of multi-plane observation mode allowed the capture of high-resolution images with an enhanced depth of field. This setup facilitated the assessment of pore morphology and distribution. Image processing and quantitative analysis were carried out using Fiji ImageJ software, version IJ 1.46r.

## 3. Results

### 3.1. Shrinkage Test

**Figure 5:**
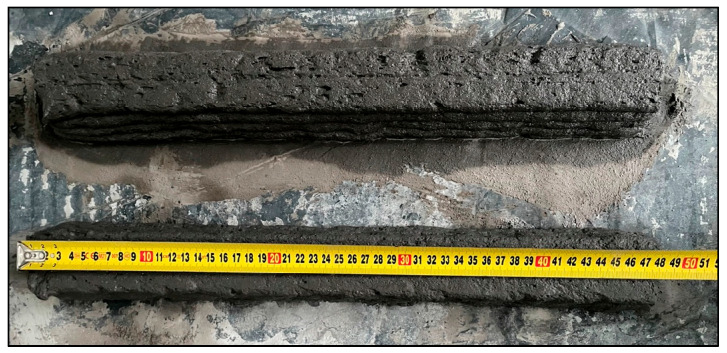
Representative view of the measurement of the sample immediately after printing.

Alkali-activated binders and geopolymers exhibit significantly higher shrinkage than ordinary Portland cement (OPC), particularly during early curing stages [64]. This behavior confirms that alkali-activated binders have significantly higher autogenous shrinkage than OPC, and that part of the shortening comes from internal reactions (self-desiccation) [65]. This is due to intense chemical reactions, low water content, and sensitivity to drying conditions factors especially critical in 3D printing applications, where structural stability and dimensional accuracy are essential. While OPC shrinkage is generally predictable and controllable with standard curing, alkali-activated binders and geopolymer shrinkage pose greater risks of cracking, delamination, and deformation in printed elements. To overcome this, mixtures require optimized compositions, controlled curing conditions, and often the use of fillers or additives to reduce shrinkage. The shrinkage behavior of the 3D-printed concrete was assessed by measuring the linear dimensional changes in the printed specimens over time. The initial length of each specimen was recorded immediately after the printing process (time 0), serving as the reference measurement (Figure 5). As illustrated in Figure 5, the foamed concrete specimens (M1–M3) and alkali-activated binder specimens (M4–M6) exhibited significantly different shrinkage behaviors, consistent with previous observations [66]. Subsequent measurements were taken after 1, 14th, and 28th day to evaluate the time-dependent shrinkage.

**Figure 6:**
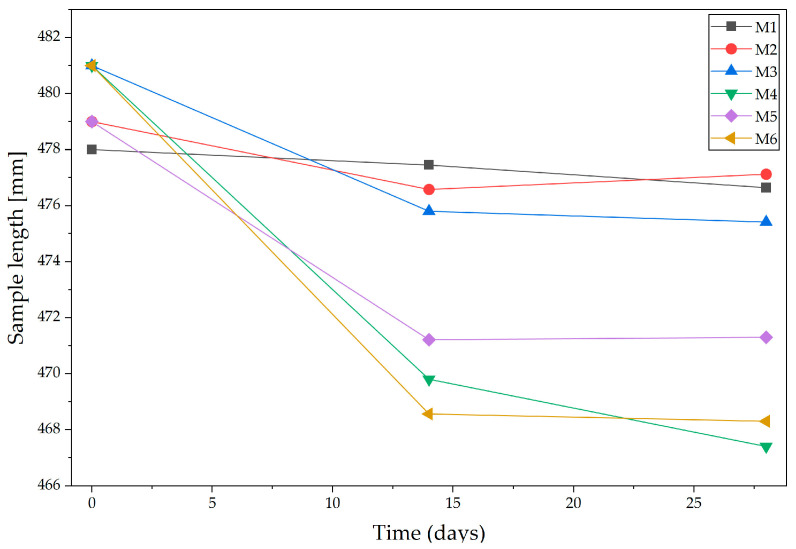
Linear shrinkage test of 3D printed samples: M1–M3 foamed concrete, M4–M6 foamed alkali-activated binder composites.

Figure 6 illustrates that the foamed concrete specimens (M1–M3) and the alkali-activated specimens (M4–M6) exhibited significantly different shrinkage behaviors, in agreement with previous findings [67]. Within the foamed concrete group, shrinkage values were relatively low, amounting after 28 days to 0.70% for M1, 0.60% for M2, and 0.96% for M3. In contrast, the alkali-activated binders displayed markedly higher shrinkage, with values after 28 days of 2.70% for M4, 1.85% for M5, and 2.50% for M6. The most favorable dimensional stability was observed in specimens containing 0.2% glass fiber and 0.3% merino fiber (M2 and M5). Conversely, M4 and M6, despite differing fiber proportions, recorded the highest shrinkage values, suggesting that in this group the matrix type exerts a stronger influence on deformation behavior than fiber composition alone. In all specimens, the majority of shrinkage occurred within the first 14 days of curing, with subsequent changes between days 14 and 28 being minimal, consistent with the observation that the greatest relative humidity decrease and gel polymerization occur during this period [68]. The results confirm that foamed concrete demonstrates superior dimensional stability compared with alkali-activated binders [68] and that the use of asymmetric fiber proportions, favoring merino fiber, can reduce shrinkage deformation in both composite types. The results further confirm that the shrinkage of alkali-activated binders or geopolymer concrete may reach values up to four times higher than those observed in conventional concrete [69,70]. The elevated shrinkage in alkali-activated binders can be explained by their microstructural evolution. Rapid reaction kinetics generate substantial internal stresses, while the relatively low water content accelerates self-desiccation, leading to autogenous shrinkage. Simultaneously, the open pore structure of these matrices enhances moisture exchange with the environment, promoting drying shrinkage. Since the present methodology relied exclusively on length change measurements, without simultaneous monitoring of mass loss or local relative humidity, it was not possible to quantitatively separate autogenous and drying contributions. Nevertheless, the observed shrinkage trends align with mechanistic models reported in the literature, where drying dominates at later stages while autogenous shrinkage is most pronounced in the first days of curing [71,72].

### 3.2. Flexural and Compressive Strength

**Figure 7:**
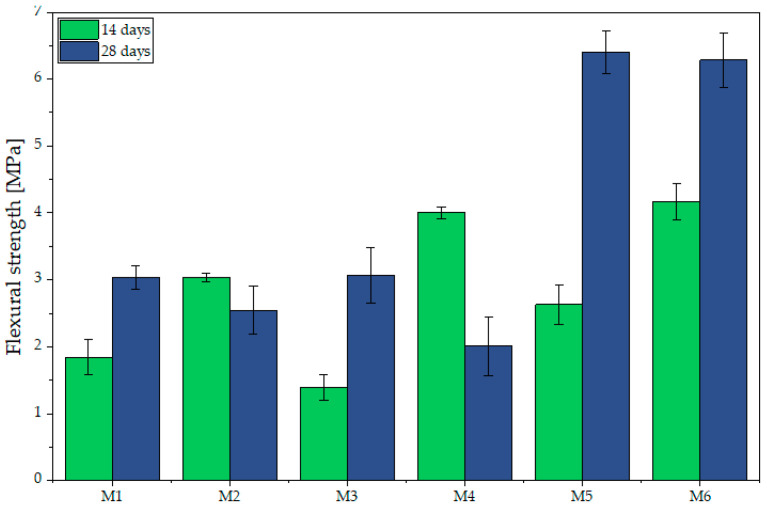
Flexural test depending on the sample composition and curing time.

The analysis of the flexural strength test results (Figure 7) indicates that, for the non-activated specimens (M1–M3), the 28-day values are moderate, ranging from 2.54 MPa (M2) to 3.03 MPa (M1) and 3.06 MPa (M3). The increase relative to the 14-day measurements is negligible, particularly in specimen M2, in which a slight decrease is even observed. In the alkali-activated specimens (M4–M6), markedly higher results were obtained, especially in M5 and M6, which after 28 days reached 6.41 MPa and 6.28 MPa, respectively. These results are consistent with the authors’ previous research [71]. Similarly, according to Alyousef et al. [73], incorporating sheep wool fibers into concrete mixtures enhanced flexural strength, thereby increasing ductility and improving the material’s capacity for energy absorption. An exception is specimen M4, which had already achieved a high strength (4.00 MPa) after 14 days but exhibited a decrease to 2.01 MPa at 28 days, potentially due to microcracking, changes in the material’s internal structure non-uniform fiber distribution. Additionally, changes in pore structure (e.g., pore coalescence or segregation of voids) could have weakened the material under bending and compressive loads [74,75]. Moreover, during 3D printing, the mixture must be fed directly to the nozzle without delays, since even short interruptions cause the mix to become denser, more difficult to extrude, and prone to reduced interlayer adhesion factors that could also contribute to the lower flexural strength observed in M4. This effect is consistent with findings by Wolfs et al. [76], who reported that increasing the time interval between printed layers reduces flexural strength due to decreased interlayer adhesion. Additionally, Lee et al. [77] showed that for 3D printed concrete members, the delay between layers correlates with lower interlayer bond strength, which in turn affects flexural behavior.

**Figure 8:**
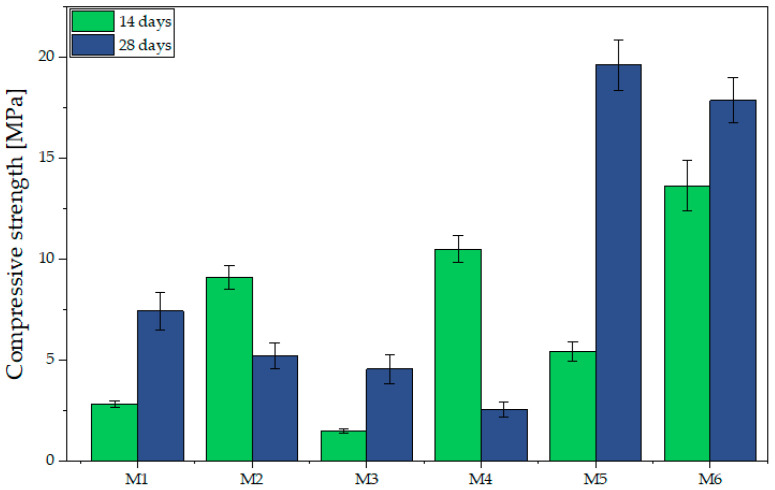
Compressive strength depending on the sample composition and curing time.

A similar trend is observed in compressive strength results (Figure 8). After 14 days, specimens M1–M3 showed low strength (M3: 1.50 MPa, M1: 2.83 MPa, M2: 9.12 MPa) with only moderate increases by day 28. In contrast, alkali-activated samples (M4–M6) achieved markedly higher values, with M5 reaching 19.63 MPa and M6 17.88 MPa after 28 days. M4 exhibited high initial strength (10.52 MPa) without further development. Gailitis et al. [78], reported similar differences for foamed concrete attained ~8.3 MPa, while geopolymer concrete reached 45–48 MPa, over five times higher. Fiber type and proportion significantly affected performance: higher merino content enhanced compressive strength (as in M5), but only in alkali-activated matrices. A 50/50 fiber ratio (M3, M6) ensured balanced properties, with M6 maintaining high strength in both flexural and compressive modes.

The analysis of the presented data in the context of the flexural-to-compressive strength ratio revealed that all tested specimens (M1–M6) considerably exceed the 15% threshold reported in the literature as indicative of materials with high resistance to flexure relative to compression [53,79]. The results range from 32.65% for M5 to 78.21% for M4, confirming the high bending load capacity of the composites. This property is advantageous for 3D printing, where layer deposition induces local bending stresses due to the absence of formwork. However, excessively high ratios, as in M4 (78.21%), may reflect low compressive strength, undesirable in load-bearing elements. For insulating applications, high ratios are beneficial, as they combine flexural resistance with moderate strength. The most favorable balance is observed in M5 (32.65%) and M6 (35.12%), which combine high ratios with very high compressive strength, ensuring strength and dimensional stability. In contrast, M1–M3 (40.67%, 48.57%, and 66.96%) may be more suitable where reduced thermal conductivity is prioritized, owing to their lower density.

### 3.3. Thermal Conductivity and Density

**Figure 9:**
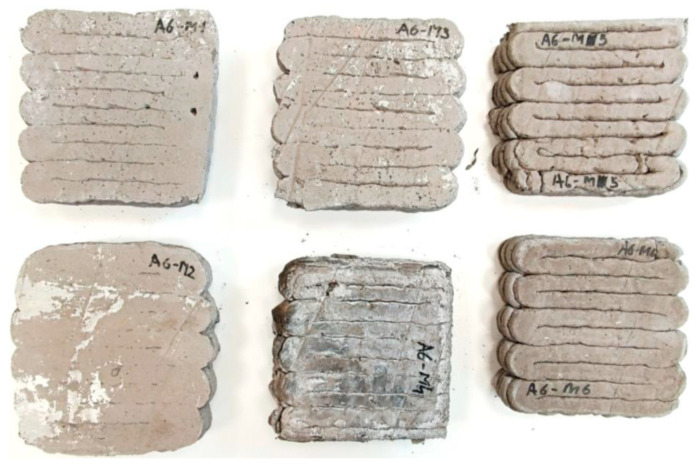
Representative view of printed samples prepared for thermal conductivity tests.

Samples of fiber-reinforced mixtures based on ordinary Portland cement (M1–M3, Figure 9) exhibit reduced thermal conductivities, ranging from 0.65 to 1.05 W/m·K, compared to typical dense concrete (Table 4). Alkali-activated binder specimens (M4–M6, Figure 9) exhibit a broader conductivity spectrum, 0.71–1.27 W/m·K. The lowest recorded value is comparable to medium-to-lightweight formulations, such as compact geopolymers (~0.43–0.47 W/m·K) and foam geopolymers (0.15–0.48 W/m·K) [80,81,82,83].

A clear dependence between density and thermal conductivity was observed (Table 4). Sample M5 and M6, with densities ≈1560–1580 kg/m^3^, correspondingly have higher conductivities (~1.06–1.27 W/m·K), while lower-density M1 and M2 (≈1330 kg/m^3^) show lower conductivities (~0.65–0.73 W/m·K), consistent with known trends in concrete systems. Moreover, fiber inclusion likely contributes to thermal transport enhancement, analogous to effects reported for hemp fibers in cementitious matrices. For example, hemp-fiber reinforcement in concrete increased thermal conductivity by ~48%, attributed to enhanced heat transfer and perhaps moisture retention [80]. It should be emphasized that, although all mixtures were foamed, their densities (1330–1580 kg/m^3^) are considerably higher than those typically reported for foamed materials (200–800 kg/m^3^). This denser pore structure results in higher λ values compared with highly porous insulating foams. Moreover, pore size distribution also plays an important role the alkali-activated specimens (M4–M6) developed a finer and less interconnected porosity compared with the non-activated mixtures containing recycled components (M1–M3), which limited the insulating effect despite the presence of pores. In addition, the presence of fibers in all samples may act as thermal bridges, further increasing conductivity. Consequently, the obtained values (0.65–1.27 W/m·K) reflect the combined effect of relatively dense microstructure, finer pore structure, and fiber reinforcement, rather than the insulating performance characteristic of low-density geopolymer foams, particularly in the case of M5 and M6.

### 3.4. Water Absorption

**Figure 10:**
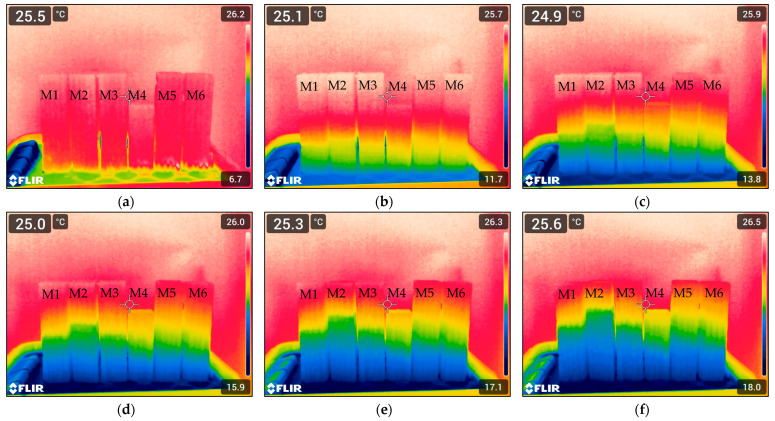
Thermal images of samples M1–M6 as a function of the duration of the water absorption test, recorded at the following intervals: (**a**) 1 min, (**b**) 50 min, (**c**) 100 min, (**d**) 150 min, (**e**) 200 min, and (**f**) 250 min.

Moisture assessment by infrared thermography (IRT) was based on surface temperature distribution, where cooler zones indicated moisture presence. Six thermograms taken at intervals between 1 and 250 min (Figure 10) clearly showed upward migration of moisture via capillary action. Since IRT does not provide direct quantification, water penetration depth was additionally evaluated from cross-sectional thermal images using Fiji ImageJ (v. IJ 1.46r). Moisture zones were delineated manually, and their lengths (in pixels) were converted into penetration depth for statistical analysis.

**Figure 11:**
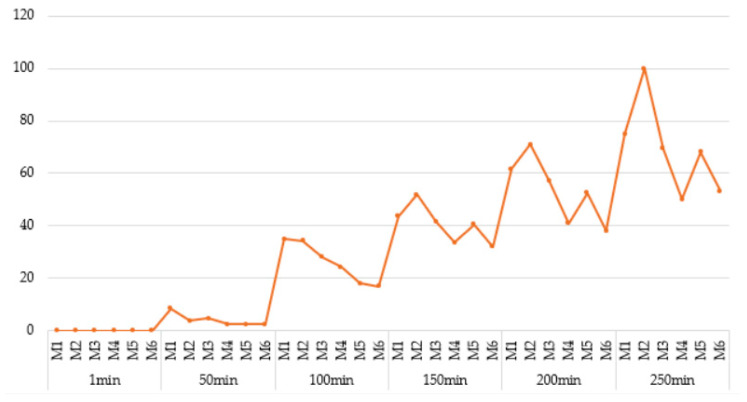
Moisture level of samples M1–M6 as a function of the duration of the water absorption test, recorded at the following intervals: 1 min, 50 min, 100 min, 150 min, 200 min, and 250 min.

As illustrated in Figure 10 and Figure 11, during the initial stage of the humidification process (t = 1 min), all specimens exhibited minimal water uptake. At this point, moisture has not yet penetrated the internal structure of the materials, and the differences between cement-based and alkali-activated-based samples were negligible. However, as early as 50 min into the exposure period, initial indications of differential moisture behavior begin to emerge. Notably, sample M2, which belongs to the non-alkali-activated cementitious group, demonstrates a significantly greater depth of water penetration relative to the other samples. In contrast, the sample with an analogous composition but alkali-activated, M5, maintains a much smaller penetration depth. This observation suggests that the alkali activation process significantly influenced the capillary penetration of water already at this relatively early stage. At 150 min, a more pronounced divergence in moisture behavior is observed. Cementitious specimens exhibit increased water penetration, whereas alkali-activated specimens maintain a relatively stable and low level of absorbency. Differences between sample pairs with identical fiber content but differing matrix types (e.g., M2 vs. M5) become increasingly evident. By the 200 min mark, this trend continues, with cementitious samples showing further increases in penetration depth, while alkali-activated samples retain their low permeability characteristics. Among all samples, M4 and M6 demonstrate the most favorable performance, exhibiting the lowest moisture penetration values throughout the observation period. The most substantial differentiation occurs at 250 min. Sample M2, which contains a higher content of merino fibers, exhibits the greatest depth of water penetration exceeding 100 mm. Conversely, its alkali-activated counterpart, sample M5, continues to demonstrate limited water ingress. A comparative analysis of samples containing 0.25% merino fibers and 0.25% glass fibers indicates that, in the case of sample M3, the balanced incorporation of hygroscopic (merino) and hydrophobic (glass) fibers can effectively mitigate the overall water uptake in cementitious matrices. This fiber composition appears to influence not only the internal pore structure and capillary connectivity but also has implications for the rheological stability and interlayer adhesion critical to extrusion-based additive manufacturing processes. Sample M6, the alkali-activated equivalent of M3, achieves the lowest recorded water penetration depth among all specimens tested, despite identical fiber content. The findings demonstrate that the incorporation of hybrid fibers resulted in a reduction in the sorption value [84], thereby indicating an enhancement in concrete quality relative to the outcomes reported in the authors’ previous investigations [85]. However, it is important to note that the production of foamed alkali-activated or geopolymer composites requires a high degree of technological precision and stringent control over parameters such as mixing sequence, component quality, temperature, and timing. These requirements render the process highly sensitive to operational variability, which may pose challenges for practical implementation under in situ construction conditions. Specifically, fluctuations in ambient environmental factors may significantly impact the moisture exchange dynamics between freshly printed materials and their surroundings, potentially affecting the long-term performance and durability of the printed elements.

A review of the literature indicates that the application of alkali activation results in the formation of a denser, microporous structure in geopolymers. This structure is characterized by a reduced number of capillary connections and significantly lower water absorption compared to materials based on Portland cement [86,87,88]. This finding is consistent with previous studies [89], which reported a significant reduction in the sorptivity of concrete following the use of geopolymer aggregate. The effect was particularly notable at 100% replacement, where a decrease in sorptivity of approximately 47.9% was observed compared to the control specimen. Furthermore, as noted by Hao et al., cementitious concretes with higher porosity and lower overall quality tend to exhibit increased water absorption and transport rates. This behavior is attributed to the presence of open and interconnected capillary pores, which facilitate the ingress of moisture [90].

### 3.5. Layer Adhesion

**Figure 12:**
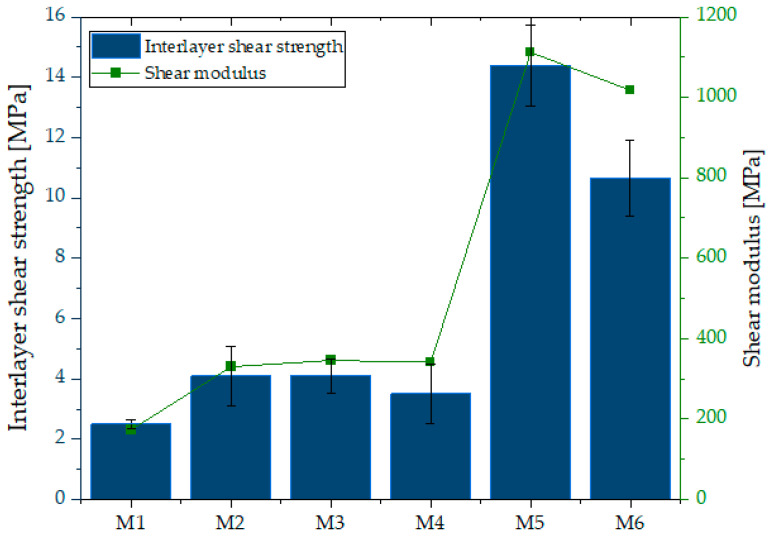
Interlayer shear strength and shear modulus of samples M1–M6.

**Figure 13:**
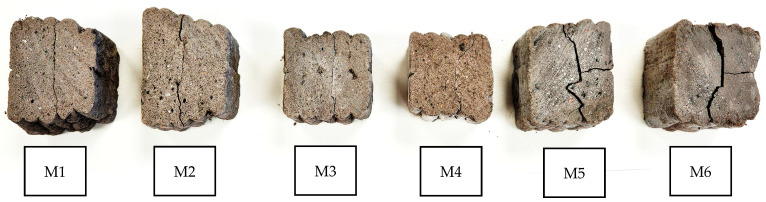
Representative view of fracture surfaces for sample M1–M6 after shear testing.

**Figure 14:**
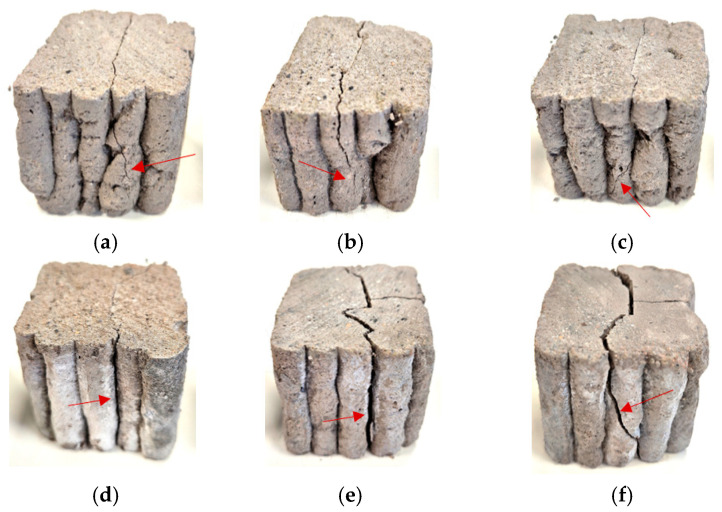
Detailed images of representative fracture surfaces of the tested samples: (**a**) M1, (**b**) M2, (**c**) M3, (**d**) M4, (**e**) M5, and (**f**) M6. Red arrows indicate the locations of dominant cracks and delamination zones.

Figure 12 demonstrates that samples M5 and M6 exhibited the highest shear strength, reaching 14.4 ± 0.6 MPa and 10.65 ± 0.8 MPa, respectively. The strength of M5 was significantly higher than that of M6 and more than four times greater than in samples M2–M4 (3.3 ± 0.2 MPa). The weakest performance was recorded for M1 (1.5 MPa). In relative terms, M5 (14.4 MPa) and M6 (10.65 MPa) demonstrated 9.6- and 7.1-fold higher shear strength compared to M1 (1.5 MPa). This substantial improvement indicates effective reinforcement of interlayer cohesion. The analysis presented on Figure 13 and Figure 14 revealed that in samples M3–M5, cracks propagated predominantly along the interlayer boundaries, indicating weaker cohesion and higher susceptibility to interfacial failure. Conversely, in M2, M5, and M6, cracks were less regular and extended over a larger portion of the sample volume, suggesting a more homogeneous material structure. Nonetheless, fiber in-corporation decreased the suspension fluidity, which in turn weakened interlayer bonding strength. Previous studies have shown that the interlayer bond strength of glass-fiber-reinforced composites is considerably lower than that of fiber-free specimens [91]. Interlayer shear strength is also strongly affected by the time gap between successive printing steps: short intervals limit the role of layer orientation, whereas prolonged pauses result in progressive bond weakening [66,92,93]. Fiber stiffness further governs cohesion—rigid glass fibers tend to align parallel to interlayer boundaries and cannot effectively penetrate interfaces [94], while more flexible fibers (merino wool) may form bridging structures that enhance adhesion. However, fiber incorporation reduces suspension fluidity, which ultimately weakens interlayer bonding [95].

In addition, a comparison of specimens with identical compositions but different activation methods clearly demonstrates the significant role of alkali activation in enhancing interlayer cohesion. For the M1–M4 pair, the shear strength increased from 1.5 MPa to 3.3 MPa (+120%). An even more pronounced improvement was observed for the M2–M5 and M3–M6 pairs, where the shear strength rose from 3.3 MPa to 14.4 MPa (+336%) and from 3.3 MPa to 10.65 MPa (+223%), respectively. These results indicate that alkali activation enables a more than three- to fourfold increase in interlayer load-bearing capacity compared to foamed concretes of analogous composition. Recent work by Luo et al. [96] demonstrated that CaO/Al_2_O_3_ and SiO_2_/Al_2_O_3_ ratios strongly affect strength, microstructural densification, and stabilization in slag/fly ash-based geopolymers. These mechanistic insights help explain why our alkali-activated mixtures (M4–M6) achieved higher interlayer shear strength, likely due to the formation of favorable gel phases and denser interfaces.

Efflorescence was observed on the surface of some specimens, particularly in the alkali-activated mixtures (Figure 14d–f). This phenomenon is associated with the migration of soluble alkali salts toward the surface during drying, followed by their crystallization upon exposure to air. Such deposits are frequently encountered in mineral-based materials when they are exposed to moisture and low ambient temperatures, especially if no protective surface coating is applied [83]. While efflorescence is primarily an aesthetic issue, its occurrence may indicate leaching processes that could influence long-term durability. Previous studies have reported that efflorescence is common in geopolymer and alkali-activated systems, especially when high alkali contents are used. In the present study, efflorescence did not interfere with mechanical testing, but its presence was documented as a secondary effect of the curing and moisture transport conditions [97,98]. Further research will be required to quantify the extent of efflorescence and evaluate its implications for the long-term performance of 3D-printed composites.

**Figure 15:**
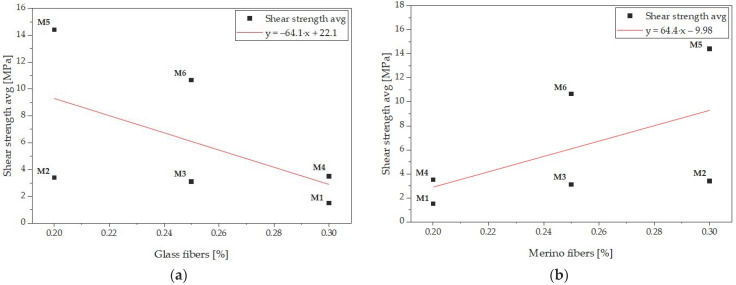
Linear regression of fiber content vs. interlayer shear strength: (**a**) glass fibers, (**b**) merino fibers.

As indicated by the regression analyses, fiber type exerts opposite effects on interlayer cohesion. Figure 15a shows a negative correlation between glass fiber content and interlayer shear strength (r = −0.55, R^2^ = 0.31). Increasing the fraction of rigid glass fibers reduces the bond quality between successive layers, which can be attributed to their high stiffness and limited deformability. These fibers tend to align parallel to the interfacial plane during extrusion and cannot effectively penetrate or interlock across the boundary, thereby acting as defects that weaken the interlayer adhesion. In contrast, Figure 15b demonstrates a moderate positive correlation between merino fiber content and interlayer shear strength (r = +0.55, R^2^ = 0.31). Unlike glass fibers, the flexible merino fibers are capable of bending and forming bridging structures that span across adjacent layers. This bridging effect enhances stress transfer across the interface, delays crack initiation, and results in a more effective reinforcement of the interlayer bond.

In practical terms, the alkali-activated mixtures M5 and M6 represent the most suitable choice in applications where maximum load-bearing capacity and minimal deformations are required. Mixtures M2 and M4 may be applied in moderately loaded structures, whereas M1 and M3 should be reserved for less demanding applications in which larger deformations and lower interlayer bond strength are acceptable.

### 3.6. Microscopy Observation

**Figure 16:**
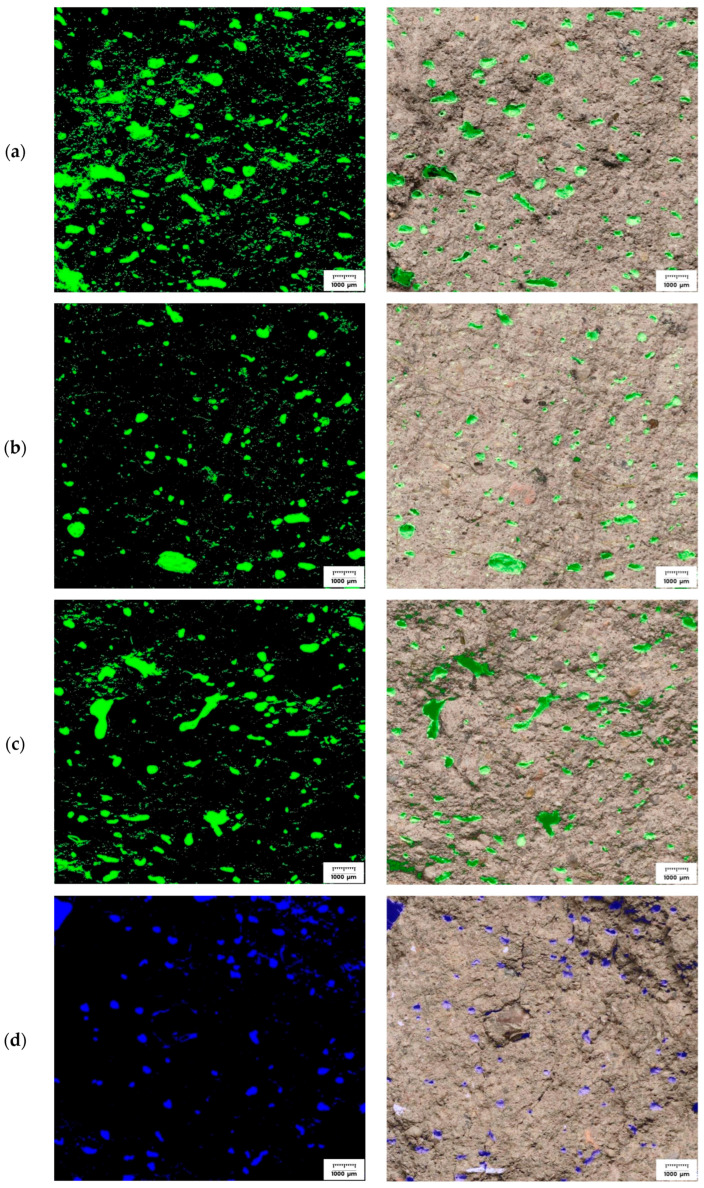

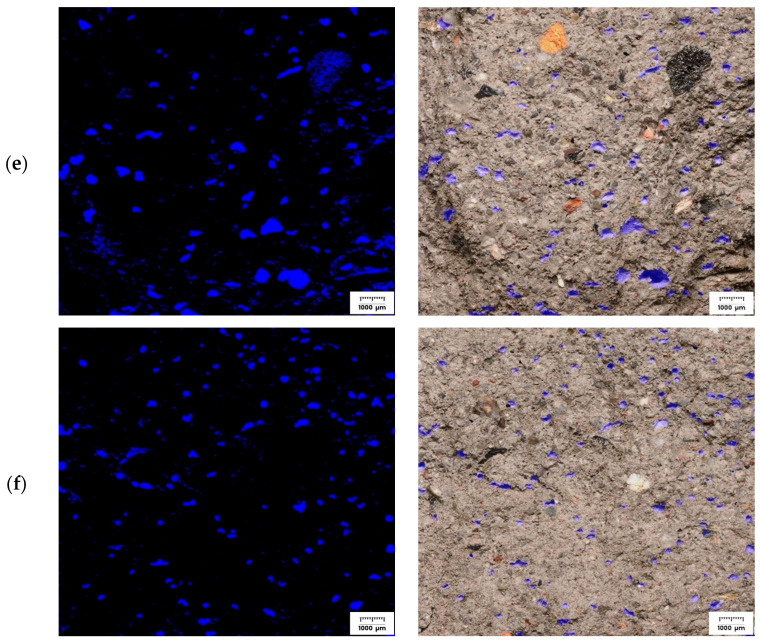
Representative microscopic images of samples: (**a**) M1, (**b**) M2, (**c**) M3, (**d**) M4, (**e**) M5, and (**f**) M6. Each set includes a fluorescence/binary contrast image (**left**) and a reflected-light image with pore markings (**right**).

In the microscopic images presented in Figure 16, revealed distinct differences between cement-based specimens (M1–M3, green) and alkali-activated binders (M4–M6, blue). Post-compression analysis confirmed that matrix type significantly influenced pore geometry. Cementitious composites (M1–M3) exhibited elongated pores and extensive capillarity, consistent with higher susceptibility to microcrack initiation. In contrast, alkali-activated specimens (M4–M6) showed lower pore elongation and a denser microstructure.

For the cementitious specimens (M1–M3), the pore structure appears more open, thereby facilitating moisture transport into the material and making it more vulnerable to water exposure compared to its alkali-activated counterparts. The alkali-activated binder specimens (M4–M6), by contrast, exhibit a denser and more closed pore structure, with a markedly reduced number of capillary connections. This structural feature corresponds with the lower values of water penetration recorded during absorption testing. In particular, specimens M5 and M6 consistently maintained low sorptivity throughout the exposure period, with M6 containing a balanced proportion of glass and merino fibers achieving the lowest penetration depth among all the tested materials.

Fiber-reinforced foamed alkali-activated composites exhibit a reduced pore density compared to foamed counterparts of identical composition without alkali activation. In these systems, the foam phase remains more stable, generating pores of smaller size and narrower distribution [99,100]. The incorporation of fibers, particularly flexible merino fibers, enhances foam stabilization [101] and promotes the formation of interlayer bridging structures [102]. However, it is only within the alkali-activated matrix that these fibers are effectively integrated into the surrounding structure, thereby reducing the development of open porosity. Consequently, foamed alkali-activated composites display a denser and less capillary pore system than their cementitious analogs, which directly translates into lower sorptivity and superior mechanical performance.

**Figure 17:**
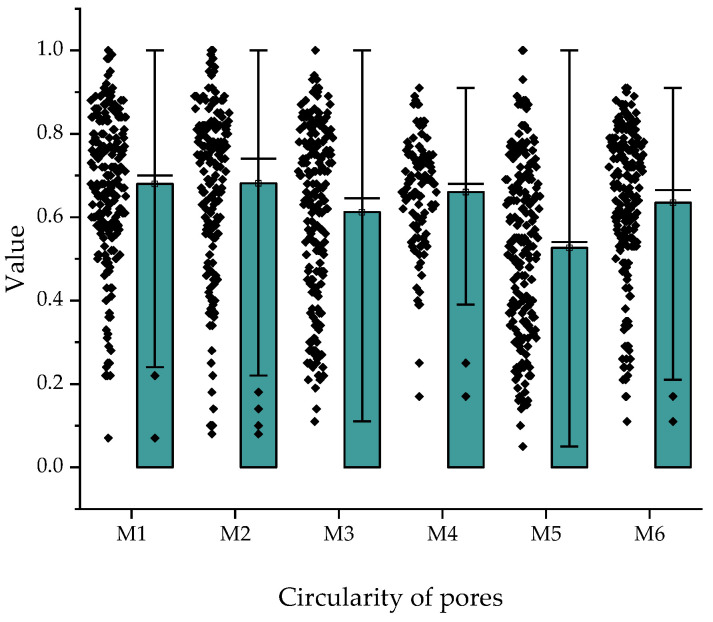
Distribution of pore circularity for mixtures M1–M6.

Pore geometry was quantified using two complementary parameters: circularity and aspect ratio. Circularity values, with 1 denoting a perfect circle, ranged between 0.5 and 0.7 across all specimens (Figure 17), indicating a predominance of irregular pore shapes. Cementitious mixtures (M1–M2) exhibited higher median circularity and lower variability, reflecting more spherical pores, whereas alkali-activated counterparts (M4–M5) showed lower medians (~0.5–0.6) and broader distributions, including markedly irregular pores. The greatest divergence was observed between M3 and M6: while M3 reached a median of ~0.6, M6 fell below 0.5 with numerous outliers below 0.2, confirming the presence of highly distorted geometries.

Aspect ratio analysis further distinguished the systems. In M1–M4, specimen M1 recorded higher median values, indicating a larger fraction of elongated pores, while M4 contained more regular, near-circular forms. A comparable pattern was found for M2–M5, where M2 exhibited higher median aspect ratio and wider dispersion than M5, which presented a tighter distribution and more uniform pore geometry. The most pronounced contrast occurred in M3–M6: M3 displayed the widest distribution, with values exceeding 5.0, evidencing severely elongated defects, whereas M6 showed a lower median and narrower spread, indicating the predominance of more regular pore morphologies.

**Figure 18:**
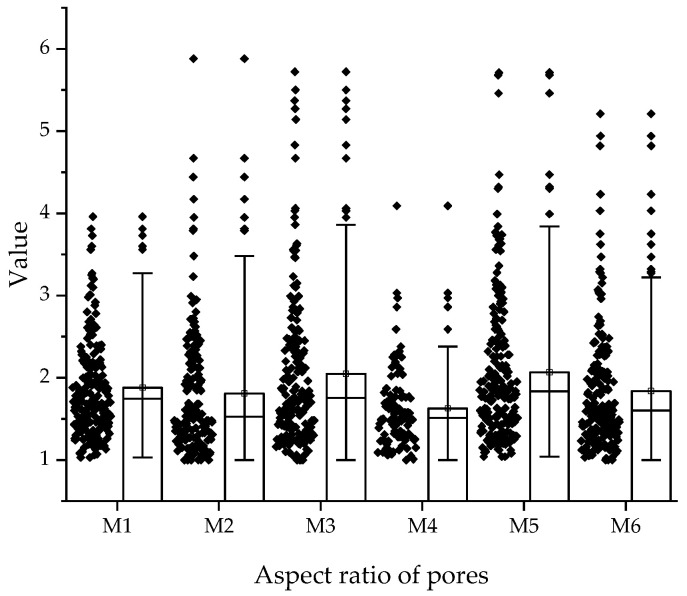
Distribution of pore aspect ratio for mixtures M1–M6.

Overall, the pore morphology analysis (Figure 17 and Figure 18) demonstrates that the principal differentiating factor among the investigated composites is not a shift in the median values of circularity or aspect ratio, but rather the reduced occurrence of extremely irregular pores. This trend is especially evident in alkali-activated mixtures (M5–M6), where the diminished presence of elongated and open capillaries contributes to improved mechanical properties and reduced water transport, as confirmed by strength and absorption tests. The comparison of specimens M3 and M6, which contained identical proportions of hybrid fibers, shows that alkali activation leads to a more stable microstructure and superior performance. Nonetheless, the non-activated variant (M3) also remains of interest for large-scale 3D printing, since the absence of alkali activation simplifies the technological process, reduces sensitivity to variable environmental conditions, and allows greater control over rheological properties during on-site application. Consequently, both activated and non-activated systems may find their specific niches: the former where maximization of strength and durability is required, and the latter where simplicity and predictability of the construction process are of greater importance.

## 4. Conclusions

This study demonstrates that the microstructural characteristics, fiber composition, and matrix type collectively govern the mechanical performance, durability, and dimensional stability of 3D-printed foamed composites. After 28 days, foamed concrete specimens (M1–M3) exhibited relatively low shrinkage values of 0.70%, 0.60%, and 0.96%, respectively, whereas alkali-activated composites (M4–M6) showed significantly higher shrinkage of 2.70%, 1.85%, and 2.50%. Moisture transport behavior further highlighted these differences: after 250 min of humidification, the cementitious specimen M2 reached the highest water penetration depth (>100 mm), while M3 achieved intermediate values due to the balanced incorporation of merino and glass fibers. By contrast, the alkali-activated counterparts M5 and M6 displayed substantially lower penetration depths, with M6 recording the lowest value among all specimens, thereby confirming the superior moisture resistance of alkali-activated hybrids. These findings were consistent with the denser and more homogeneous microstructures observed in alkali-activated composites, which correlated with enhanced mechanical strength and reduced water absorption compared to cement-based mixtures. Nevertheless, non-activated foamed concretes, particularly M3, remain attractive for large-scale 3D printing, as their simpler processing and lower sensitivity to environmental variability present practical advantages for on-site construction despite lower mechanical performance. 

## Figures and Tables

**Table 1:** The chemical composition of ground demolition brick.

Oxide composition (%)	MgO	Al_2_O_3_	SiO_2_	SO_3_	Cl	K_2_O
	3.32%	23.64%	58.03%	0.22%	0.10%	3.95%
	CaO	TiO_2_	Cr_2_O_3_	MnO	Fe_2_O_3_	ZrO_2_
	1.99%	1.06%	0.09%	0.06%	7.00%	0.29%

**Table 2:** Fiber characteristics.

Fibers	Diameter [µm]	Length [mm]	Density [g/cm^3^]
Glass (G)	20.00	6 mm	2.50
Merino wool (M)	18.20	6 mm	4.55

**Table 3:** Composition of mixtures and their designation.

No.	Designation of the Mixtures	w/c	Cement [%]	Sand [%]	Fly Ash [%]	Coal Slag [%]	Ground Bricks [%]	Glass Fibers [%]	Merino Fibers [%]
1	M1	0.34	27	37	5	15	15	0.3	0.2
2	M2	0.34	27	37	5	15	15	0.2	0.3
3	M3	0.34	27	37	5	15	15	0.25	0.25
4	M4	0.34	27	37	5	15	15	0.3	0.2
5	M5	0.34	27	37	5	15	15	0.2	0.3
6	M6	0.34	27	37	5	15	15	0.25	0.25

**Table 4:** Density, thermal conductivity, and thermal resistance depending on the composition of the samples.

Designation of Sample	Density (kg/m^3^)	λ (W/m·K)	R (m^2^·K/W)
M1	1351	0.65	0.074
M2	1330	0.73	0.067
M3	1387	1.05	0.049
M4	1431	0.71	0.069
M5	1561	1.27	0.038
M6	1580	1.06	0.045

## Data Availability

The original contributions presented in this study are included in the article. Further inquiries can be directed to the corresponding author.
